# Rab17 mediates differential antigen sorting following efferocytosis and phagocytosis

**DOI:** 10.1038/cddis.2016.431

**Published:** 2016-12-22

**Authors:** Charles Yin, Yohan Kim, Dean Argintaru, Bryan Heit

**Affiliations:** 1 Department of Microbiology and Immunology and The Centre for Human Immunology, The University of Western Ontario, Schulich School of Medicine and Dentistry, London, ON, Canada N6A 5C1

## Abstract

Macrophages engulf and destroy pathogens (phagocytosis) and apoptotic cells (efferocytosis), and can subsequently initiate adaptive immune responses by presenting antigens derived from engulfed materials. Both phagocytosis and efferocytosis share a common degradative pathway in which the target is engulfed into a membrane-bound vesicle, respectively, termed the phagosome and efferosome, where they are degraded by sequential fusion with endosomes and lysosomes. Despite this shared maturation pathway, macrophages are immunogenic following phagocytosis but not efferocytosis, indicating that differential processing or trafficking of antigens must occur. Mass spectrometry and immunofluorescence microscopy of efferosomes and phagosomes in macrophages demonstrated that efferosomes lacked the proteins required for antigen presentation and instead recruited the recycling regulator Rab17. As a result, degraded materials from efferosomes bypassed the MHC class II loading compartment via the recycling endosome – a process not observed in phagosomes. Combined, these results indicate that macrophages prevent presentation of apoptotic cell-derived antigens by preferentially trafficking efferocytosed, but not phagocytosed, materials away from the MHC class II loading compartment via the recycling endosome pathway.

Clearance of apoptotic cells in a timely and efficient manner is essential for preventing the induction of inflammation and the maintenance of homeostasis.^1, 2^ The process of apoptotic cell clearance, termed efferocytosis, is performed by both professional phagocytes such as macrophages^3, 4^ and dendritic cells,^5, 6^ and by some non-phagocytic cell types such as epithelial cells.^7, 8^ Apoptotic cells that are not cleared undergo secondary necrosis, driving inflammation and autoimmunity through the release of self-antigens and proinflammatory intracellular contents into the extracellular milieu.^9^ Efferocytosis is a particularly important physiological function of macrophages, with defective macrophage efferocytosis implicated in the development of a number autoimmune and inflammatory disorders including systemic lupus erythematosus^10, 11, 12^ and atherosclerosis.^6, 13, 14, 15, 16^ Despite recognition of the importance of efferocytosis in the maintenance of homeostasis, the mechanisms regulating efferocytosis remain poorly elucidated. It is thought that the molecular mechanisms of efferocytosis resembles those of phagocytosis, with the important distinction that the ultimate outcome of phagocytosis is the induction of inflammation and antigen presentation, whereas efferocytosis is immunologically silent.^2, 10, 17^ While a number of ligands, mediators and receptors that regulate efferocytosis have been identified and characterized,^10^ little is known of the maturation process that degrades efferocytosed cells, or the processes that determine the ultimate fate of degraded apoptotic cells.

In phagocytosis, the phagosome maturation process responsible for the degradation of engulfed bacteria is well characterized (reviewed in Flannagan *et al.*^18^), and the maturation of efferosomes appears to share much of the same cellular machinery.^19^ In both processes the target microbe or apoptotic cell is internalized into a plasma membrane-derived vacuole, respectively, termed the phagosome and efferosome, where these engulfed targets are degraded. The earliest maturation event following the internalization of phagocytic and efferocytic cargos is the recruitment of the small GTPase Rab5 to the nascent phagosome or efferosome, a process driven by the Rab5 GTPase-activating protein Gapex-5.^20^ Once activated, Rab5 mediates the fusion of early endosomes to the phagosome or efferosome via the Rab5 effectors EEA-1, Vps-34 and Mon1a/b.^19, 21^ Within minutes of engulfment Rab5 is exchanged for Rab7, upon which Rab7 then mediates the fusion of late endosomes and lysosomes with the phagosome or efferosome. While this latter portion of the efferocytosis pathway has not been fully characterized, it is believed to proceed in a manner identical to that of phagocytosis. In phagocytosis, inactive GDP-bound Rab7 is exchanged for Rab5 by a complex of Mon1a/b and Ccz-1, and is then activated by the exchange of GDP for GTP, a process induced by either Ccz-1 or the HOPS complex.^21, 22, 23^ Active Rab7, through effectors such as RILP and ORPL1, coordinates the movement of lysosomes along microtubules and their subsequent fusion with the phagosome, thereby delivering the vacuolar ATPase and catabolic enzymes that mediate the degradation of phagocytosed materials.^24, 25, 26^ Ultimately, the phagolysosome containing this degraded phagocytosed material matures into a tubular lysosome in which the loading of MHC class II with phagocytosed antigens occurs.^27^

The consequence of phagosome maturation is the induction of both inflammatory signaling and antigen presentation by the phagocyte.^28, 29, 30^ In contrast, efferosome maturation is known to be anti-inflammatory and non-immunogenic. Immediately following the efferocytic uptake of apoptotic cells, PPAR*γ* and the atypical chemokine receptor D6 initiate signaling, which suppresses early inflammatory processes such as the oxidative burst.^4, 31, 32, 33^ This suppression of inflammation is maintained over intermediary periods of time through transcriptional processes initiated by these receptors. The upregulation of mIR-21 following efferocytosis inhibits inflammatory signaling by silencing PDCD4 and PI3K, resulting in decreased TNF*α* expression through p38 MAPK-dependent signaling, while simultaneously enhancing IL-10 production and further uptake of apoptotic cells through increased PI3K/AP-1 signaling.^34, 35^ In addition to inducing mIR-21, continued signaling through PPAR*γ* directly drives the expression of anti-inflammatory cytokines including IL-10 and TGF*β*.^33, 35^ This anti-inflammatory state can be maintained over prolonged periods of time through the polarization of macrophages to an anti-inflammatory/pro-efferocytic M2 or M2c state.^7, 36^ While these processes contribute to creating an anti-inflammatory environment, it remains unclear how immunogenicity is avoided following efferocytosis. Tissue-resident macrophages constitutively express MHC class II and costimulatory molecules, as do *in vitro* unpolarized (M0) and M2-polarized macrophages.^37, 38, 39^ Indeed, M2 macrophages are fully competent to present antigens, and can induce naive CD4+ T cells to differentiate into Th17 effectors.^40^ Clearly, additional mechanisms must be in place to prevent the immunogenic presentation of apoptotic cell-derived antigens. In this study, we tested the hypothesis that efferocytosed apoptotic cells undergo a novel maturation process that bypasses the MHC class II loading compartment. Using mass spectrometry and fluorescence microscopy, we identified a Rab17-dependent maturation process that mediates the transfer of degraded apoptotic cell materials to the recycling endosome and away from the MHC class II loading compartment, thereby preventing apoptotic cell-derived materials from intersecting the macrophage antigen presentation machinery.

## Results

### Efferosomes and phagosomes share a common early maturation pathway

**Figure 1:**
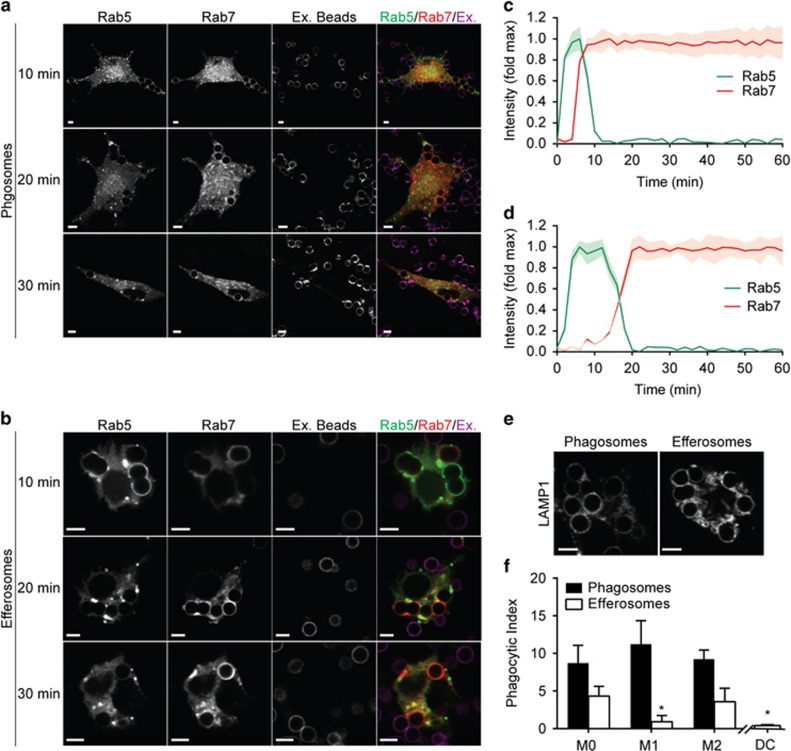
Efferosomes and phagosomes share a common early maturation pathway. Fixed and live cell microscopy was used to assess the localization of Rab5, Rab7 and the lysosomal marker LAMP1 to efferosomes and phagosomes containing 5 *μ*m diameter bead-based mimics of apoptotic cells and immunoglobulin G (IgG)-opsonized pathogens. (**a** and **b**) Recruitment of Rab5 and Rab7 to phagosomes (a) and efferosomes (b) 10, 20 and 30 min following engulfment by J774.2 macrophages. Ex. Beads indicates non-internalized (i.e. extracellular) beads. (**c** and **d**) Dynamics of Rab5 and Rab7 recruitment to phagosomes (**c**) and efferosomes (**d**). *T*=0 is set as the video frame when complete sealing of the phagosome/efferosome was observed; data are normalized to maximum Rab5 or Rab7 intensity on each individual phagosome. (**e**) Immunostaining of LAMP1 accumulation in phagosomes or efferosomes in human peripheral blood mononuclear cell (PBMC)-derived M0 macrophages. (**f**) Uptake of apoptotic cell mimics (Efferosomes) and IgG-opsonized pathogen mimics (Phagosomes) by human PBMC-derived M0-, M1- and M2-polarized macrophages and DCs, expressed as the number of beads engulfed per cell. Data are representative of (**a**, **b** and **e**) or quantifies (**c**, **d** and **f**) at least 30 cells imaged across five independent experiments. Data are presented as mean or mean±S.E.M. *P<0.05 compared with uptake of the same type of target by M0 macrophages. Scale bars are 5 *μ*m

Phagocytic cargos are degraded by a well-characterized maturation pathway in which the sequential acquisition of Rab5 and Rab7 mediates the sequential fusion of early endosomes, late endosomes and lysosomes with the phagosome.^18^ Evidence from *Caenorhabditis elegans* and mammalian cell lines indicate that this same maturation pathway degrades efferocytosed apoptotic cells.^19^ However, these studies did not use professional antigen-presenting cells (pAPC), which may use an alternative pathway to avoid antigen presentation following uptake of apoptotic cells. To test whether apoptotic cells were trafficked through a novel maturation pathway in pAPCs, we tracked the recruitment of ectopically expressed Rab5-GFP and Rab7-RFP in J774.2 macrophages engaged in phagocytosis (Figures 1a and c) or efferocytosis (Figures 1b and d) of 5 *μ*m diameter synthetic phagocytic or efferocytic targets. Both efferosomes and phagosomes sequentially recruited Rab5 and Rab7, with efferosomes transitioning from a Rab5-positive to Rab7-positive compartment with slightly slower temporal dynamics than phagosomes (Figures 1c and d). To confirm that both phagosomes and efferosomes were completing maturation, we immunostained human PBMC-derived M0 macrophages for the lysosomal marker LAMP1 40 min after engulfment of synthetic phagocytic or efferocytic targets (Figure 1e). Significant LAMP1 accumulation was observed on phagosomes and efferosomes, confirming that vacuoles containing both types of cargo fuse with lysosomes. Last, we assessed the possibility that macrophage polarization may result in the selective uptake of phagocytic *versus* efferocytic targets (Figure 1f). M0 and M2 macrophages were highly efferocytic, while M1 macrophages and primary human dendritic cells were poorly efferocytic. Furthermore, no selectivity was observed for phagocytic targets, indicating that any capacity to differentially present efferosomal *versus* phagosomal antigens must occur following both target engulfment and the canonical Rab5/Rab7-mediated maturation pathway.

### Mass spectrometric identification of late regulators of efferosome and phagosome maturation

Given that efferosomes and phagosomes shared the same early maturation pathway, any selective processing of these targets likely occurs at a later time point. As such, we recovered efferosomes and phagosomes using 3 *μ*m diameter magnetic bead mimics of efferocytic and phagocytic targets from M0-polarized human macrophages, 40 min after initiation of efferocytosis or phagocytosis. Proteins from the recovered efferosomes and phagosomes were resolved using SDS-PAGE, revealing a number of proteins selectively recruited to efferosomes *versus* phagosomes (Supplementary Figure 1). These unique proteins were subsequently excised and identified by liquid-chromatography/mass spectrometry. Phagosomes (Table 1) contained many of the proteins expected of a vesicle maturing into an MHC class II loading compartment, notably MHC class II and indicators of Golgi-to-lysosome trafficking (Rab6b, PIK4). In addition, phagosomes were enriched in a number of proteins involved in GTPase and kinase signaling. In marked contrast, efferosomes (Table 2) lacked MHC class II and the markers of Golgi-to-lysosome trafficking observed on phagosomes. Efferosomes instead recruited proteins that mediate vesicular trafficking, cytoskeletal organization and ubiquitination/ISG15ylation (Table 2). Of particular interest were the Rab-family GTPases Rab17 and RASEF (Rab45), both of which have been implicated in trafficking to recycling endosomes and exocytosis.^41, 42, 43, 44^ Combined, these results suggest that efferocytic cargos are intercepted before formation of the MHC class II loading compartment and are redirected to a recycling or exocytic cellular compartment.

### Rab17 is persistently recruited to efferosomes but not phagosomes

**Figure 2:**
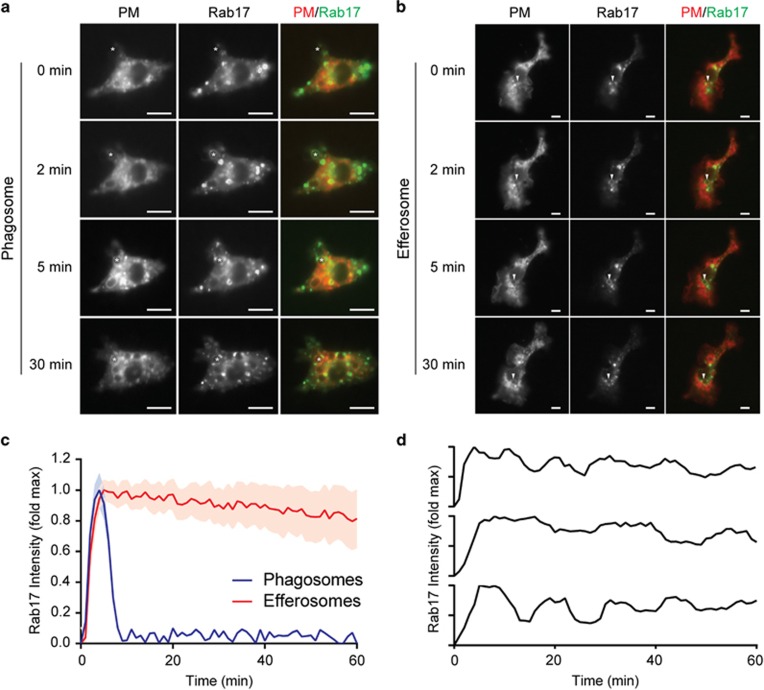
Rab17 is selectively retained on efferosomes. Live cell microscopy was performed on J774.2 macrophages expressing PM-RFP and Rab17-GFP engulfing apoptotic cell (Efferosomes) or immunoglobulin G (IgG)-opsonized pathogen (Phagosomes) mimics. (**a** and **b**) Representative images of Rab17-GFP recruitment to phagosomes (**a**) or efferosomes (**b**). */Arrowhead tracks the same phagosome/efferosome through successive time points. (**c**) Dynamics of Rab17 recruitment to phagosomes and efferosomes. (**d**) Recruitment dynamics of Rab17-GFP to three representative efferosomes. (**c** and **d**) *T*=0 is set as the video frame when complete sealing of the phagosome/efferosome was observed and data are normalized to maximum Rab17 intensity on each individual phagosome. Data are presented as mean±S.E.M. (**c**) or mean (**d**). Data are representative of (**a**, **b** and **d**) or quantifies (**c**) a minimum of 22 cells imaged over four independent experiments. Scale bars are 5 *μ*m

To assess the role of Rab17 in phagocytosis and efferocytosis, we performed live cell microscopy of J774.2 macrophages ectopically expressing the plasma membrane marker PM-RFP and Rab17-GFP as they engulfed beads mimicking efferocytic or phagocytic targets. As expected, both phagocytic and efferocytic targets were internalized into plasma membrane-derived vacuoles demarcated by PM-RFP (Figures 2a and b). Rab17 transiently localized to phagosomes, with dynamics similar to that of Rab5 (Figures 2a and c). In marked contrast, Rab17 was persistently recruited to efferosomes, displaying only a modest decrease in recruitment over the hour following engulfment of the efferocytic target (Figures 2b and c). Close inspection of individual efferosomes revealed that this recruitment was not constant, and rather that Rab17 is repeatedly recruited in a series of waves, suggestive of repeat sampling of efferosomes by Rab17 (Figure 2d). Rab17 expression was observed in primary human PBMC-derived M0-, M1- and M2-polarized macrophages as well as primary human PBMC-derived DCs, with late recruitment of Rab17 observed to efferosomes in M0- and M2-polarized macrophages and DCs (Supplementary Figure 2). RASEF and Rab6b, the other Rab-family GTPases identified in our mass spectrometry screen, did not significantly associate with either phagosomes or efferosomes (data not shown).

**Figure 3:**
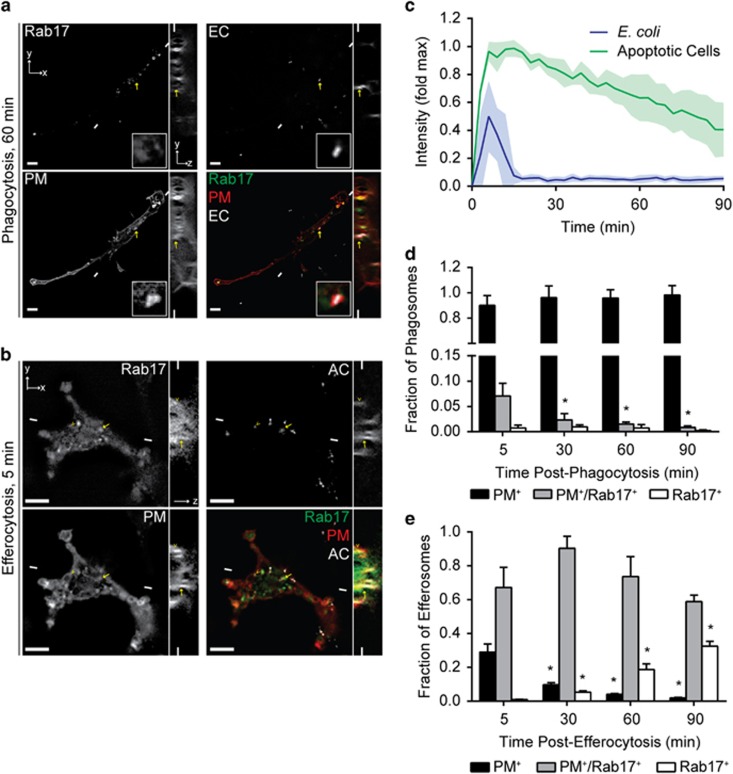
Rab17 is selectively retained on apoptotic cell containing efferosomes. Live cell microscopy was performed on J774.2 macrophages expressing PM-RFP and Rab17-GFP engulfing fluorescently tagged apoptotic cells (AC) or *E. coli* (*E. coli* or EC). (**a**) Representative slice from a z-stack of Rab17-GFP and PM-RFP recruitment to *E. coli* containing phagosomes 60 min following phagocytosis. Arrow indicates the phagosome magnified in the insert. Insert is 4 *μ*m × 4 *μ*m. (**b**) Representative slice of a z-stack of Rab17-GFP and PM-RFP recruitment to an apoptotic cell containing efferosome 5 min following efferocytosis. Arrow indicates the same Rab17^+^/PM-RFP^+^ efferosome, and arrowhead indicates the same Rab17^−^/PM-RFP^+^ efferosome, in all panels. (**c**) Recruitment dynamics of Rab17-GFP to phagosomes and efferosomes, normalized to the brightest Rab17^+^ cell structure. (**d** and **e**) Portion of *E. coli* containing phagosomes (**d**) or apoptotic cell containing efferosomes (**e**), which are positive for PM-RFP only (PM^+^), positive for PM-RFP and Rab17 (PM^+^/Rab17^+^) or positive for Rab17 alone (Rab17^+^). Scale bars are 5 *μ*m, small lines indicate the position of the cross-section shown in the corresponding *xy* and *z* images. (**c**–**e**) *T*=0 is set as the video frame when complete sealing of the phagosome/efferosome was observed, and data are normalized to maximum Rab17 intensity observed in each cell and is presented as mean±S.E.M. Data are representative of (**a** and **b**) or quantifies (**c**–**e**) a minimum of 52 cells imaged over four independent experiments. *P<0.05 compared with the 5 min time-point for the same group

Because our model system used non-digestible mimics of bacterial and apoptotic cells, we could not assess whether Rab17 altered the trafficking of degraded materials derived from efferosomes or phagosomes. As such, we performed live cell imaging of Rab17-GFP- and PM-RFP-expressing macrophages as they engulfed *Escherichia coli* or apoptotic cells covalently labeled with a degradation-resistant, pH-stable far-red fluorophore. Bacterial phagocytosis did not differ from the mimics, with *E. coli* internalized into a compartment that transiently colocalized with Rab17 and retained PM-RFP for at least 90 min (Figures 3a, c and d). In marked contrast, macrophages efferocytosed small fragments (apoptotic bodies) from apoptotic cells into PM-RFP-demarcated efferosomes, on which Rab17 was retained for prolonged periods of time (Figures 3b, c and e). Interestingly, at later time points, degraded apoptotic cell materials were observed to move into a compartment negative for PM-RFP (Figure 3d), a phenomenon not observed with non-degradable targets (Figure 2b), suggesting that degraded efferocytosed materials were being directed out of the canonical maturation pathway – a process not observed with *E. coli* (Figure 3e), indicating that the degraded apoptotic cells and *E. coli* had been trafficked into different cellular compartments.

### Rab17 mediates the trafficking of degraded apoptotic cell materials to the recycling endosome

**Figure 4:**
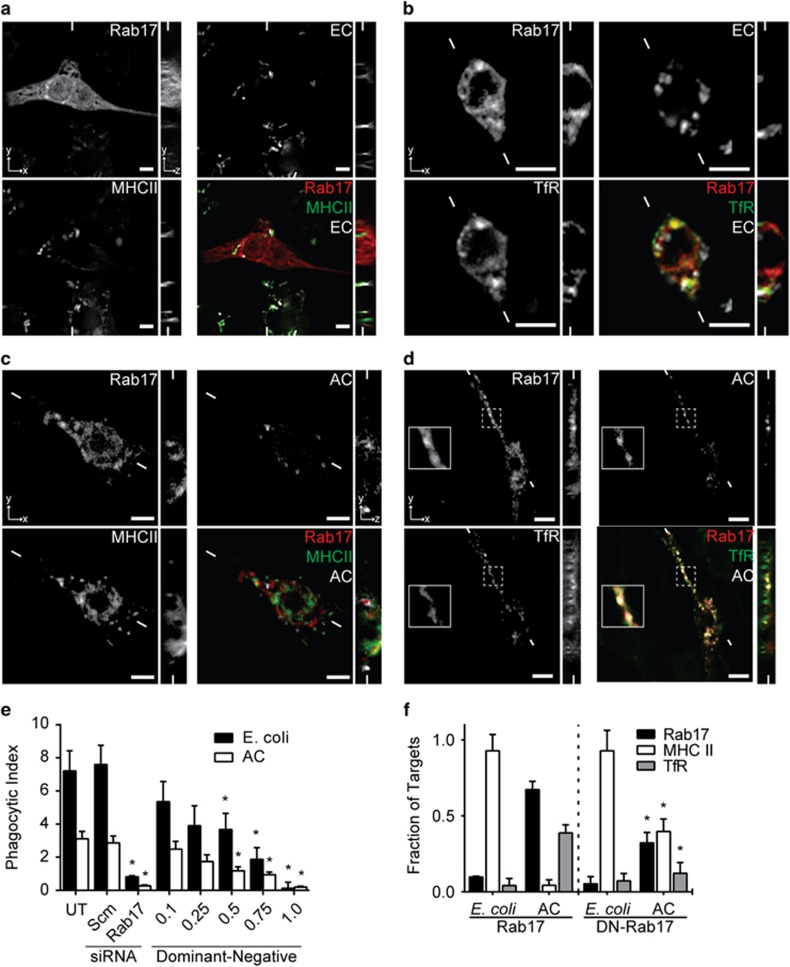
Rab17 mediates trafficking of degraded apoptotic cell materials. Z-stacks were captured of fixed J774.2 macrophages expressing Rab17-mCherry and either coexpressing TfR-GFP (TfR-GFP) or immunostained for MHC class II 90 min following engulfment of *E. coli* (EC) or apoptotic cells (AC). (**a–d**) Localization of Rab17 and MHC class II (**a** and **c**) or TfR (**b** and **d**), relative to phagocytosed *E. coli* (**c** and **d**) or efferocytosed apoptotic cells (**a** and **b**). Insert is 4 *μ*m × 4 *μ*m. (**e**) Uptake of *E. coli* and apoptotic cells by J774.2 macrophages that were untreated/non-transfected (UT), treated with scrambled (Scm) or Rab17 siRNA (Rab17), or transfected with 0.1–1.0 *μ*g per well of a construct expressing dominant-negative Rab17, expressed as the number of *E. coli* or apoptotic cells engulfed per cell. (**f**) Quantification of the fraction of phagocytosed *E. coli* or efferocytosed apoptotic cells that recruited Rab17, MHC class II and TfR in cells expressing mCherry-tagged wild-type or dominant-negative (DN) Rab17. Images are representative of, and graphs quantify, a minimum of 15 images captured in three independent experiments. Scale bars are 5 *μ*m, and small lines indicate the position of the cross-section shown in the corresponding *xy* and *z* images. *P<0.05 compared with the same group in untreated cells (**e**) or cells expressing wild-type Rab17 (**f**)

The previous data are consistent with a model in which phagocytosed materials traffic through the canonical maturation pathway, whereas efferocytosed materials are redirected into another cellular compartment by Rab17. Given that Rab17 has previously been found to regulate recycling and exocytosis, it was likely that efferocytosed materials were being trafficked from the efferosome to the recycling endosome.^41, 42^ We therefore assessed the recruitment of Rab17, a recycling endosome marker (transferrin receptor, TfR) and MHC class II to phagosomes and efferosomes. Consistent with the canonical maturation process, 90 min following engulfment most phagosomes had recruited MHC class II but not Rab17 or TfR (Figures 4a and b). In contrast, efferocytosed materials did not colocalize with MHC class II, which remained diffusively distributed throughout the cell (Figure 4c). Instead, efferosomes strongly colocalized with TfR, with a portion colocalizing with both Rab17 and TfR (Figure 4d). We next attempted to knockdown Rab17, but transfection with siRNA concentrations that reliably knocked down Rab17 expression also inhibited phagocytosis and efferocytosis (Figure 4e). Instead, we transfected J774.2 cells with a mCherry-tagged dominant-negative Rab17 at a dose that inhibited ~50% of efferocytosis and phagocytosis (0.5 *μ*g per well; Figure 4e). Dominant-negative Rab17 was not recruited to phagosomes, and was recruited to a significantly lower fraction of efferosomes compared with wild-type Rab17 (Figure 4f and Supplementary Figure 3). Importantly, expression of dominant-negative Rab17 significantly increased the association of MHC class II, and decreased the association of TfR, with efferosomes (Figure 4f and Supplementary Figures 3c and d). Combined, these results indicate that Rab17 is selectively recruited to efferosomes, where it mediates the transfer of degraded apoptotic cell material into the recycling endosome and away from the MHC class II loading compartment.

## Discussion

Macrophages are tasked with the engulfment and destruction of both apoptotic cells and pathogens, and following engulfment must, respectively, generate immunologically silent *versus* immunogenic responses. While several processes that enable macrophages to suppress inflammatory responses following engulfment of apoptotic cells have been reported,^4, 7, 31, 32, 33, 34, 35, 36^ identification of a mechanism preventing antigen presentation following efferocytosis, but not phagocytosis, remains elusive. In this study, we have identified a Rab17-dependent pathway in which macrophages redirect degraded apoptotic cells away from the MHC class II loading compartment and towards the recycling endosome, where disruption of this pathway resulted in degraded apoptotic cell materials reaching an MHC class II-positive intracellular compartment. This mechanism appears to be present in dendritic cells, as well as macrophages, both of which are capable of presenting phagocytosed antigens on MHC class II. Although previous studies have demonstrated a need for TLR or cytokine signaling to induce the formation of an MHC class II loading compartment and loading of phagosome-derived antigens onto MHC class II,^27, 45^ the selective recruitment of Rab17 to efferosomes and its exclusion from phagosomes occurred in the absence of these signals, suggesting that this pathway is intrinsic to efferocytic receptor signaling and independent of TLR/cytokine-induced formation of the MHC loading compartment.

Rab17 function is best understood in polarized epithelial cells where it is required for both receptor recycling through the apical recycling compartment and for receptor-mediated transcytosis.^42, 43^ Disrupting Rab17 in polarized epithelial cells resulted in the inappropriate basolateral-to-apical transcytosis of transferrin and an accumulation of normally basolateral proteins on the apical facet of the cell.^42, 46^ While the role of Rab17 in non-polarized cells is not completely elucidated, it is known to localize to the recycling endosome,^42^ and is required for the exocytic processes driving both the release of melanosomes from melanocytes and the extension of dendrites from hippocampal neurons.^41, 47, 48^ Moreover, Rab17 is required for the formation of bactericidal autophagosomes following Group A *Streptococci* invasion of epithelial cells.^49^ Our observation that Rab17 is required for the trafficking of apoptotic cell-derived materials from the phagolysosome to the recycling endosome is consistent with Rab17's role in mediating traffic through the recycling endosome, and while it was not observed in this study, it is possible that the capacity of Rab17 to regulate exocytosis may mediate the expulsion of degraded apoptotic cell materials from the macrophage. At this time, the role of Rab17 in other immune cells has not been investigated. However, the portion of the maturation process regulated by the sequential acquisition of Rab5 and Rab7 components has been observed to function in a nearly identical manner following phagocytosis in macrophages, DCs and neutrophils,^50^ and following efferocytosis in macrophages and DCs.^19^ Moreover, we have observed Rab17 recruitment to efferosomes late in the maturation process in murine macrophage cell lines, primary human macrophages and primary human DCs, indicating that Rab17 likely acts to prevent antigen presentation in both macrophages and dendritic cells. In addition, Rab17 expression has been reported in other efferocytic cell types, including epithelial cells.^41, 43, 46, 49^ Although epithelial cells do not normally present antigens on MHC class II, the presence of Rab17 in these cells suggests that they may sort apoptotic cell-derived materials via the recycling endosome, perhaps expelling degraded apoptotic cell materials from the apical cell surface.^43^

Rab17 is known to be recruited and activated by Rabex-5, a guanine exchange factor that can recruit and activate both Rab17 and Rab5,^47, 51^ and indeed, this pathway likely accounts for the transient recruitment of Rab17 to phagosomes observed in this study. This initial wave of Rab17 may be involved in the rapid recycling of phagocytic and efferocytic receptors from the nascent phagosome and efferosome, a process required to maintain phagocytic and efferocytic capacity by returning receptors to the cell surface.^52, 53^ Indeed, the initial wave of Rab17 recruitment and the deleterious effect of dominant-negative Rab17 expression on phagocytosis and efferocytosis that we observed is consistent with the established role of receptor recycling in phagocytosis. Previous studies have shown that Rab11-mediated recycling is required for maximal phagocytic capacity in macrophages, with the expression of dominant-negative Rab11 greatly reducing phagocytosis through Fcγ receptors.^54^ Defective Rab11 signaling resulted in the trapping of receptors in the recycling endosome, thereby impairing further activity through sequestering receptors from the cell surface.^55^ Rab17 is known to mediate the recycling of other membrane receptors such as the transferrin, pIgR and Fc-like receptors,^42, 43^ indicating a theoretical capacity to mediate the recycling of efferocytic and phagocytic receptors. Indeed, we did not observe any phagocytic receptors, and only weakly detected two efferocytic receptors (CD36 and Galectin-3^56, 57^), in phagosomes and efferosomes recovered late in the maturation process. Furthermore, Rab17 knockdown or expression of dominant-negative Rab17 decreased both phagocytosis and efferocytosis, consistent with the need for ongoing receptor recycling to maintain phagocytic capacity.^54^ Combined, these data suggest that early Rab17 recruitment recycles receptors from the nascent phagosome or efferosome, and is required for the maintenance of phagocytic/efferocytic capacity of macrophages.

While Rabex-5 mediated recruitment may explain the initial wave of Rab17 observed on phagosomes, it is unlikely to be involved in the repeated waves of Rab17 recruitment observed throughout efferosome maturation. Indeed, the Rab7 adaptor Mon1 is known to displace Rabex-5 from the phagosome,^58^ and our results indicate that Rab17 sampling of the efferosome temporally overlaps with Rab7 recruitment to the efferosome. Another Rab GTPase, Rab27, can be localized to the phagosome,^59^ activate Rab17^41^ and drive exocytosis,^60, 61^ indicating that it may be a candidate for the recruitment of Rab17 to the efferosome. However, we were unable to identify Rab27 on efferosomes in either our mass spectrometry screen or by immunofluorescence (data not shown), although the sensitivity and temporal limitations of these methods may have prevented identification of transient or weak Rab27 recruitment. The enrichment of ubiquitination/ISG15ylation machinery on the efferosome identified in our mass spectrometry screen may indicate a third possible mechanism for Rab17 recruitment to efferosomes. Rab17 is known to require monosumoylation, a ubiquitin-like post-translational modification, for its recruitment and activity.^46^ The presence of molecular machinery, including E3 ligases that modify proteins with ubiquitin and ISG15 motifs, may indicate that Rab17 recruitment and activation on efferosomes may occur through a SUMO-like post-translational modification driven by efferosome-resident E3 ligases.

The sorting of apoptotic cell-derived antigens away from the MHC class II loading compartment is likely an important component of peripheral tolerance, as the 60 to 80 billion apoptotic cells generated daily in the human body represents a significant antigenic burden dealt with predominantly by resident phagocytes.^62^ Unsurprisingly, defects in efferocytic receptors and signaling molecules are associated with the onset of autoimmune disorders including rheumatoid arthritis, multiple sclerosis and systemic lupus erythematosus,^11, 63, 64^ and with chronic inflammatory conditions such as atherosclerosis.^13, 15, 65^ However, this regulation must be more nuanced than simply sorting all degraded apoptotic cells away from the MHC class II loading compartment, as apoptotic cells can harbor pathogens and act as vehicles for pathogen dissemination.^66^ Furthermore, MHC class II stability and trafficking to MHC class II loading compartments is enhanced following TLR or cytokine stimulation of dendritic cells, suggesting that inflammatory signaling may act to enhance trafficking and loading of MHC class II following phagocytosis.^67, 68^ The effects of a proinflammatory microenvironment or the activation of TLR/cytokine signaling on Rab17-mediated trafficking of efferosomal contents away from the MHC class II compartment remains to be characterized.

In summary, we have delineated a Rab17-dependent pathway that allows phagocytes to selectively divert engulfed and degraded apoptotic cells away from the MHC class II loading compartment and into the recycling endosome, thereby limiting the potential for presenting autoantigens. These findings suggest that the differential sorting of apoptotic cell-derived *versus* pathogen-derived antigens is a hereto unappreciated mechanism involved in maintaining peripheral tolerance.

## Materials and methods

### Materials

J774.2 macrophages, PM-RFP, Rab5-GFP, Rab7-mCherry and TfR-GFP expression constructs were gifts from Dr. Sergio Grinstein (Hospital for Sick Children, Toronto, ON, Canada). *E. coli* DH5*α* and ML35 were gifts from Drs. John McCormick and Susan Koval, respectively (University of Western Ontario, London, ON, Canada). Human Rab17 cDNA was purchased from the Harvard PlasmID Repository (Boston, MA, USA). RPMI, DMEM, Trypsin-EDTA and fetal bovine serum (FBS) were purchased from Wisent (St. Bruno, QC, Canada). Recombinant human M-CSF, GM-CSF, INF*γ* and IL-4 were purchased from Peprotech (Rocky Hill, NJ, USA). No. 1.5 thickness round coverslips and 16% paraformaldehyde (PFA) were purchased from Electron Microscopy Supplies (Hatfield, PA, USA). Colloidal Coomassie Blue was purchased from Bio-Rad (Mississauga, ON, Canada). Lympholyte-poly, all fluorescent secondary antibodies and fluorescently labeled streptavidin were purchased from Cedarlane Laboratories (Burlington, ON, Canada). Anti-mouse MHC class II and Cell Proliferation Dye eFluor 670 were purchased from eBioscience (San Diego, CA, USA). Phosphatidylserine (PtdSer), phosphatidylcholine (PtdChol) and biotin-phosphatidylethanolamine (biotin-PE) were purchased from Avanti Polar Lipids (Alabaster, AL, USA). *Salmonella* lipopolysaccharide and human IgG were purchased from Sigma-Aldrich (St. Louis, MS, USA). Anti-human LAMP1 was purchased from the Developmental Studies Hybridoma Bank (Iowa City, IA, USA). Phusion DNA polymerase, FugeneHD, dithiobis[succinimidyl propionate], dithio-bismaleimidoethane, Permafluor and ON-TARGETplus siRNA were purchased from ThermoFisher (Mississauga, ON, Canada). DNA oligos were purchased from IDT (Coralville, IA, USA). Restriction enzymes and T4 DNA ligase were purchased from New England Biolabs (Whitby, ON, USA). Silica and silica-functionalized magnetic beads of 3–5 *μ*m were purchased from Bangs Laboratories (Fishers, IN, USA). Anti-Rab17 was purchased from Proteintech (Chicago, IL, USA). All other chemicals were purchased from Canada BioShop (Mississauga, ON, Canada).

### J774.2 macrophage culture

J774.2 murine macrophages were cultured in T25 tissue culture flasks containing 5 ml of DMEM buffered with sodium bicarbonate and supplemented with 10% FBS. Cells were grown to 80% confluency at 37 °C in a 5% CO_2_ incubator and passaged by cell scraping. Cells were cultured for no more than six generations, at which time new cells were revived from liquid nitrogen stocks. For microscopy experiments, no. 1.5 thickness, 18 mm diameter round coverslips were placed into the wells of a 12-well tissue culture plate, 1 ml of DMEM+10% FBS added to each wells and 0.2 ml (~2.5 × 10^5^ cells per well) of the above cell suspension added to each well. Cells were cultured for at least 8 h before transfection. Transfection with DNA vectors or siRNA was conducted using FugeneHD, as per the manufacturer's instructions. Briefly, for each well in a 12-well plate, 1.1 *μ*g of DNA or 0.75 *μ*g of siRNA was diluted into 100 *μ*l of serum-free DMEM, followed by 3.3 *μ*l of FugeneHD. The resulting mixture was incubated for 20 min at room temperature, and then added dropwise to the J774.2 cells. Cells were incubated for at least 18 h at 37 °C in a 5% CO_2_ before imaging.

### Human macrophage and DC culture

The collection of blood from healthy donors was approved by the Health Science Research Ethics Board of the University of Western Ontario and venipuncture was performed in accordance with the guidelines of the Tri-Council Policy Statement on human research. Blood was drawn into heparinized vacuum collection tubes, layered on an equal volume of Lympholyte-poly and centrifuged at 300 × *g* for 35 min at 20 °C. The top band of peripheral blood mononuclear cells was collected, washed once (300 × *g*, 6 min, 20 °C) with phosphate-buffered saline (PBS, 137 mM NaCl, 2.7 mM KCl, 10 mM Na_2_HPO_4_, 1.8 mM KH_2_PO_4_), and the cell pellet was suspended in RPMI-1640+10% FBS and 1% antibiotic–antimycotic solution at a density of ~2 × 10^6^ cells per ml. Monocytes were separated by adhesion to glass by placing 200 *μ*l or 3.6 ml of the cell suspension, respectively, on sterile 18 mm coverslips or 100 mm diameter gelatin-coated tissue culture plates, and non-adherent cells removed after a 1 h/37 °C incubation with two gentle washes of warmed PBS. Monocytes were then differentiated into M0-, M1- or M2-polarized macrophages by culturing for 5 days in 10 ng/ml M-CSF (M0 and M2) or 20 ng/ml GM-CSF (M1). After 5 days, the media were replaced with DMEM+10% FBS supplemented with 10ng/ml M-CSF (M0), 20 ng/ml GM-CSF+250 ng/ml *Salmonella* lipopolysaccharide+10 ng/ml INF*γ* (M1), or with 10 ng/ml M-CSF+10 ng/ml IL-4 (M2) and cultured an additional 2 days. Cells were used between days 7 to 10 of culture. To produce DCs, monocytes were cultured for 48 h with 100 ng/ml GM-CSF and 100 ng/ml IL-4. Cells were used immediately following differentiation.

### Preparation of synthetic phagocytic and efferocytic targets

Synthetic targets were prepared as per our previously published methods.^69^ Briefly, for imaging experiments 5 *μ*m silica beads, and for mass spectrometry experiments 3 *μ*m silica-functionalized magnetic beads, were coated with lipids or IgG to produce beads that mimic apoptotic cells or bacteria, respectively. Apoptotic cell membrane mimics were prepared by preparing 0.4 mmol of a lipid solution comprised of 19.8% PtdSer, 80% PtdChol and 0.2% biotin-PE into a glass vial. Control cell mimics were prepared using a lipid mixture comprised of 99.8% PtdChol and 0.2% biotin-PE. Ten microliters of the silica or silica-magnetic beads were added to the lipid mixture, vortexed for 30 s and the chloroform evaporated under a flow of nitrogen for 1 h. The resulting bead mixture was suspended in 1 ml of PBS, and washed 3 × by pelleting the beads using a 4500 × *g*/1 min centrifugation, aspiration of the supernatant and resuspension in 1 ml of PBS. Beads were suspended in 100 *μ*l of PBS after the final wash. IgG-coated beads were prepared by washing 10 *μ*l of the silica or silica-magnetic beads 3 × in PBS, as described above. Beads were then suspended in 100 *μ*l of PBS+10 *μ*l of a 50 mg/ml human or murine IgG solution and incubated for 60 min at room temperature with continuous mixing. Beads were then washed 3 × in PBS and suspended in a final volume of 100 *μ*l of PBS, as described above.

### Preparation of apoptotic cells and heat-killed *E. coli*

Jurkat T cells were cultured in T25 flasks containing 5 ml of RPMI-1640+10% FBS, and split 1:10 into fresh media every 5 days. To induce apoptosis, 1 ml of cultured cells were pelleted with a 300 × *g*/5 min centrifugation and resuspended in 1 ml of serum-free RPMI+1 *μ*M staurosporine. Cells were incubated for 3 to 12 h at 37 °C, washed three times with PBS and used within 4 h of preparation. If required, apoptotic cells in PBS were fluorescently labeled by adding 2 *μ*l/ml Cell Proliferation Dye eFluor 670 for 5 min at room temperature, followed by washing 3 × in PBS. ML35 *E. coli* were grown to stationary phase in LB media at 37 °C with shaking at 200 r.p.m., and 0.1 ml (~1 × 10^7^ bacteria) of the culture was pelleted by a 1500 × *g*/1 min centrifugation and resuspended in 0.1 ml of PBS. The bacteria were then heat killed by incubating for 10 min at 70 °C, followed by incubation with 0.5 *μ*l/ml Cell Proliferation Dye eFluor 670 for 5 min at room temperature. Excess dye was quenched by the addition of 0.9 ml of fresh LB media and the cells were washed 3 × in PBS. After washing, the cells were opsonized by resuspending the bacteria in 1 ml of DMEM+10% human serum, incubating at 37 °C for 10 min, washed once in PBS and resuspended in 0.1 ml of PBS. Bacteria were used within 24 h of preparation.

### Mass spectrometry

For mass spectrometry assays, 1.8 × 10^7^ (~60 *μ*l) of silica-magnetic bead mimics were added to 100 mm tissue culture plates containing M0-polarized human macrophages. Targets were mixed into the plates with gentle shaking, forced into contact with the macrophages by a 1 min/250 × *g* centrifugation and then incubated at 37 °C in 5% CO_2_ for 40 min. The samples were then washed vigorously with PBS+1 mM EDTA to remove non-internalized beads, and phagosome/efferosome-bound proteins crosslinked to the beads using the ReCLIP method.^70^ Briefly, after washing PBS+ReCLIP reagent (0.5 mM dithiobis[succinimidyl propionate] and 0.5 mM dithio-bismaleimidoethane) was added to the cells and incubated for 20 min at room temperature. The ReCLIP reagent was then aspirated and crosslinking stopped by the addition of 10 ml of quenching buffer (5 mM l-cysteine+20 mM Tris-Cl, pH 7.4) for 10 min. Cells were suspended in lysis buffer (250 mM sucrose, 10 mM HEPES, 3 mM MgCl, 1 mM NaVO_4_, 1:50 DNAse I, 0.25 mM PMSF, 200 nM okadaic acid, 10 mM NaF, phosSTOP and 1 × completeMini phosphatase/protease inhibitors), transferred to an ice-cold nitrogen cavitator and lysed using a 10 min, 300 PSI cavitation. Efferosomes and phagosomes were then recovered using a magnetic column, washed 3 × in lysis buffer and solubilized for 30 min at 37 °C in 30 *μ*l of 1 × Laemmli buffer+50 mM DTT and 1 × phosSTOP/completeMini phosphatase/protease inhibitors.

Recovered efferosomal and phagosomal proteins were boiled for 5 min, loaded onto a precast 4–20% gradient SDS-PAGE gel and separated for 3 h at 100 V. Gels were then fixed for 2 h in 40% ethanol+10% glacial acetic acid, incubated overnight with colloidal Coomassie Blue and destained in ddH_2_O for 3 h with gentle agitation. Gels were stored in ddH_2_O at 4 °C before mass spectrometry. Protein bands unique to the phagosome or efferosome lanes were then picked using an Ettan Robotic Spot-Picker (GE Health Sciences, Baie d'Urfè, QU, Canada). Gel slices were destained with 50 mM ammonium bicarbonate and 50% acetonitrile, treated with 10 mM DTT and 55 mM iodoacetamide, and digested with trypsin. Peptides were extracted by a solution of 1% formic acid and 2% acetonitrile and freeze dried. Dried peptide samples were then suspended in a solution of 10% acetonitrile and 0.1% trifluoroacetic acid. Mass spectrometry analysis of each sample was performed using an AB Sciex 5800 TOF/TOF System (Framingham, MA, USA). Raw mass spectrometry data analysis was performed using MASCOT database search (http://www.matrixscience.com).

### Rab17 cloning

Human Rab17 was PCR amplified (forward primer: 5′-AAAAAAAAAGAATTCGATGGCACAGGCACACAGGACCCC-3′ reverse primer: 5′-TTTTTTTTTGGATCCCTAGTGGGCGCAGCATTTGGCCT-3′) using Phusion polymerase as per the manufacturer's instructions with 35 cycles of denaturation (30 s, 98 °C), annealing (30 s at 61 °C) and extension (1 min at 72 °C). The resulting amplicon was gel purified and digested with *Eco*RI and *Bam*HI for 1 h at 37 °C. The restriction fragment as well as *Eco*RI/*Bam*HI-digested pEGFP-C1 was recovered using a PCR cleanup kit as per the manufacturer's instructions and ligated overnight at 16 °C with T4 ligase. The resulting construct was transformed into chemically competent DH5*α*, grown on LB agar containing 50 *μ*g/ml kanamycin and insertion confirmed by Sanger sequencing performed by the London Regional Genomics Centre (London, ON, Canada). Dominant-negative (N132I) Rab17 constructs^46^ were prepared by amplifying the above construct with 5′ phosphorylated primers (5′-GCCCACCAGCATCACCAGGACTTC-3′ and 5′-ATCAAGACGGACCTCAGCCAGGAGCGG-3′) using the same PCR cycle as above, except with a 7 min elongation time. Parental plasmid was removed by the addition of *Dpn*I (1 : 50 (vol/vol)) to the PCR reaction and incubating for 1 h at 37 °C. The resulting PCR product was then gel purified, recircularized by incubating overnight at 16 °C with T4 ligase, transformed into DH5*α* and recovered using LB-kanamycin plates. Mutation was confirmed by Sanger sequencing.

### Efferocytosis/phagocytosis assays

For microscopy-based assays ~1 × 10^6^ of the efferocytic or phagocytic targets were added to each well of a 12-well plate (~3 *μ*l of bead mimics or ~10 *μ*l of apoptotic cells or heat-killed *E. coli*). Targets were mixed into the wells by gentle shaking and forced into contact with the macrophages by a 1 min, 250 × *g* centrifugation. For live cell microscopy experiments, cell-target mixtures were kept at 10 °C until imaged, whereas samples for fixed-cell imaging were immediately returned to a 5% CO_2_ incubator at 37 °C. For live cell imaging experiments, coverslips were transferred to a Leiden chamber filled with 37 °C imaging buffer (150 mM NaCl, 5 mM KCl, 1 mM MgCl_2_, 100 *μ*M EGTA, 2 mM CaCl_2_, 20 mM HEPES, 1.5 g/l NaHCO_3_) and the Leiden chamber placed onto the heated/CO_2_-perfused live cell piezoelectric stage of a Leica DMI6000B microscope equipped with a × 100/1.40 NA objective, photometrics Evolve-512 delta EM-CCD camera, Chroma Sedat Quad filter set and the Leica Application Suite X software platform (Leica Microsystems, Concord, ON, Canada). The positions of 5–15 transfected cells were marked using the mark and find feature, and live cell acquisitions of each marked cells acquired at 0.5–1 frames per min, with recordings conducted for 60–120 min per coverslip. For fixed-cell imaging, cells were incubated at 37 °C/5% CO_2_ for the desired time following target addition, washed once with room temperature PBS, and if required, non-internalized beads labeled by the addition of 1 *μ*l/ml Alexa-647-labeled streptavidin or 1:1000 Alexa-647-labeled anti-human/mouse Fab. Cells were then fixed for 20 min at room temperature with PBS+4% PFA and then blocked for 60 min using PBS+5% human serum or 5% bovine serum albumin; if permeabilized cells were required, 0.1% Triton X-100 was added during this step. Proteins of interest were labeled following blocking/permeabilization by the addition of primary antibodies (1:1000 anti-MHC class II, 1:500 anti-LAMP1, 1:200 anti-Rab17) followed by secondary fluorescently tagged Fab's (1:000 to 1:2500) for 1 h in blocking buffer, with 3 × 15 min washes in PBS performed after each antibody incubation. All labeling and washing was performed at room temperature. Labeled samples were mounted on glass coverslips using Permafluor mounting media, and a minimum of 15 cells imaged on each coverslip.

### Image analysis

All image analysis was performed in MATLAB (MathWorks, Natick, MA, USA) using modified forms of our previously published vesicle-tracking and intensity quantification algorithms.^71, 72, 73^ Briefly, 90–120 min time-lapse videos were captured of macrophages ectopically expressing the plasmalamellar marker PM-RFP, GFP-tagged Rab17 and far-red-labeled targets for phagocytosis/efferocytosis. TIFF stacks of individual cells were then exported and a local threshold applied to the target channel using a local neighborhood 2 × the size of the targets. Targets were then identified using the 'bwlabel' command in MATLAB and the targets tracked using a robust tracking algorithm.^72, 74^ Image channels corresponding to PM-RFP and Rab17 were subjected to a global background subtraction, with no other processing applied, to maintain linearity of intensity data. Internalized targets were defined as targets that colocalized with a distinct PM-RFP structure that was no more than 1.4 × (beads) or 2 × (*E. coli* or apoptotic cells) the diameter of the phagocytic/efferocytic target, and the first frame in which the target colocalized with a distinct PM-RFP structure was set as the 0 min time point of phagocytosis/efferocytosis. All tracks were manually curated to ensure accuracy, with any tracks lasting <15 min rejected from subsequent analyses. Recruitment of Rab17 to the PM-RFP/target structure was then measured at all subsequent time points, and normalized to the maximum intensity Rab17 structure in the cell.

Colocalization of signaling molecules with phagosomes and efferosomes in fixed cells was quantified using a similar technique. Z-stack images of cells were captured and deconvolved using an iterative deconvolution approach. A region of interest (ROI) was manually drawn around each cell and the mean±S.E.M. of the fluorescence intensity of each channel within the ROI determined. Phagocytic/efferocytic targets were then identified using the local background subtraction and thresholding approach described above, but using the 'bwlabeln' MATLAB command to identify targets in the 3D images. Each target was converted into a 3D ROIs by identifying the bounding pixels of each target using the 'convhull' command. These ROIs were then expanded by a factor of 1.4 ×, which was determined experimentally to increase ROI size such that the bounding PM-RFP and any recruited signaling molecules would be captured without significant inclusion of cytosolic staining (data not shown). Targets were scored as associated with a molecule of interest if the mean intensity of the molecule of interest in each ROI was 2 S.D. higher than the mean intensity of the molecule throughout the entire cell. As with the live cell analyses, no intensity adjustments other than a global background subtraction were applied to the images of the signaling molecules to preserve linearity of the image intensity data.

### Statistics

GraphPad Prism software (GraphPad Software, La Jolla, CA, USA) was used for all statistical tests. Unless otherwise noted, ANOVA with Tukey's correction was used for analysis. Data are presented as mean±S.E.M. *P*-values ≤0.05 are considered to be significant and are indicated by an asterisk.

## Figures and Tables

**Table 1:** Unique proteins recruited to phagosomes 40 min post-phagocytosis

**Protein name**	**Symbol**	**Peptides**^a^	**% Coverage**^b^
*Vesicular trafficking and antigen presentation*			
MHC class I	MR1	62	34.3
MHC class II	HLADQB1	58	22.2
Annexin A2	ANXA2	57	19.9
Phosphatidylinositol-4-kinase	PIK4	56	20.1
Rab6B	Rab6b	83	39.9
CTTNBP2NL	CTTNBP2NL	204	31

*GTPase and kinase signaling*			
Insulin-like growth factor binding protein 2	IGFBP2	126	38.8
Misshapen-like kinase 1	MINK1	91	7.01
Cyclin-Y-like protein 2	CCNYL2	58	33.3
Lymphoid enhancer-binding factor 1	LEF1	89	29.4
RalA-binding protein 1	RALBP1	82	17.7
Serine/threonine-protein kinase 4	STK4	29	10.6
NOD3	NLRC3	111	15.5
5′-AMP-activated protein kinase *β*1	PRKAB1	15	6.05
GIMD1	GIMD1	24	11.1
Cyclin-dependent kinase 4	CDK4	20	18.0
DOCK10	DOCK10	58	10.7

*Other*			
Acylphosphatase-1	ACYP1	37	43.5
Cytotoxic T-lymphocyte protein 4	CTLA4	46	26.4
Type I inositol(1,4,5)trisphosphate 5 phosphatase	INPP5A	30	96.8
Abhydrolase domain containing 1	ABHD1	63	33.9
Lactate dehydrogenase c	LDHC	41	42.7
Striatin, calmodulin-binding protein 3	STRN3	48	10.8
CKLF-like MARVEL transmembrane domain-containing protein 1	CMTM1	14	11.5
Interferon-stimulated 20 kDa exonuclease-like 2	ISG20L2	12	8.70

a Peptides=total number of peptides from each protein identified across three independent experiments.

b % Coverage=portion of protein sequence covered by the identified peptides

**Table 2:** Unique proteins recruited to efferosomes 40 min post-phagocytosis

**Protein name**	**Symbol**	**Peptides**^a^	**% Coverage**^b^
*Vesicular trafficking and cytoskeleton*			
Rab17	Rab17	69	36.3
RASEF (Rab45)	RASEF	32	72.7
Vacuolar protein sorting-associated protein 33B	VPS33B	95	16.6
Talin 1	TLN1	198	7.79
Rac2	RAC2	19	12.8
MAP4	MAP4	119	13.9
Liprin *β*1	PPFIBP1	86	31.6
Prostaglandin F2 receptor-negative regulator	PTGFRN	89	11.3
WAS/WASL-interacting protein family member 3	WIPF3	48	14.7

*Ubiquitination/ISG15ylation*			
E3 ubiquitin/ISG15 ligase TRIM25	TRIM25	85	20.1
Ubiquitin carboxyl-terminal hydrolase 15	USP15	115	12.1
F-box-only protein 6	FBXO6	74	21.9

*Kinase and calcium signaling*			
Serine/threonine-protein phosphatase 2A regulatory subunit B	PPP2RC3	46	10.2
MAP kinase-interacting serine/threonine-protein kinase 1	MKNK1	12	5.29
Calsyntenin 2	CLSTN2	49	8.06
Glycogen synthase kinase-3*β*	GSK3B	18	100

*Receptors and opsonins*			
Galectin-3	LGALS3	69	47.3
Mannan-binding lectin serine protease 1	MASP1	13	15.5
CD36	SCARB1	18	58.1

*Other*			
Leucine-rich repeat neuronal protein 1	LRRN1	48	57.1
Inositol monophosphatase 2	IMPA2	72	27.6
Alkaline phosphatase, tissue-nonspecific isozyme	ALPL	10	66.7

a Peptides=total number of peptides from each protein identified across 3 independent experiments.

b % Coverage=portion of protein sequence covered by the identified peptides

## References

[bib1] Korns D, Frasch SC, Fernandez-Boyanapalli R, Henson PM, Bratton DL. Modulation of macrophage efferocytosis in inflammation. Front Immunol 2011; 2: 1–10. 22566847 10.3389/fimmu.2011.00057 PMC3342042

[bib2] Green DR, Oguin TH, Martinez J. The clearance of dying cells: table for two. Cell Death Differ 2016;: 1–12. 10.1038/cdd.2015.172 PMC4987729 26990661

[bib3] Tao H, Yancey PG, Babaev VR, Blakemore JL, Zhang Y, Ding L et al. Macrophage SR-BI mediates efferocytosis via Src/PI3K/Rac1 signaling and reduces atherosclerotic lesion necrosis. J Lipid Res 2015; 56: 1449–1460. 26059978 10.1194/jlr.M056689 PMC4513986

[bib4] Pashover-Schallinger E, Aswad M, Schif-Zuck S, Shapiro H, Singer P, Ariel A. The atypical chemokine receptor D6 controls macrophage efferocytosis and cytokine secretion during the resolution of inflammation. FASEB J 2012; 26: 3891–3900. 22651933 10.1096/fj.11-194894

[bib5] Thorp E, Subramanian M, Tabas I. The role of macrophages and dendritic cells in the clearance of apoptotic cells in advanced atherosclerosis. Eur J Immunol 2011; 41: 2515–2518. 21952808 10.1002/eji.201141719 PMC3289088

[bib6] Thorp EB. Mechanisms of failed apoptotic cell clearance by phagocyte subsets in cardiovascular disease. Apoptosis 2010; 15: 1124–1136. 20552278 10.1007/s10495-010-0516-6 PMC3744319

[bib7] Stanford JC, Young C, Hicks D, Owens P, Williams A, Vaught DB et al. Efferocytosis produces a prometastatic landscape during postpartum mammary gland involution. J Clin Invest 2014; 124: 4737–4752. 25250573 10.1172/JCI76375 PMC4347249

[bib8] Ichimura T, Asseldonk EJPV, Humphreys BD, Gunaratnam L, Duffield JS, Bonventre JV. Kidney injury molecule-1 is a phosphatidylserine receptor that confers a phagocytic phenotype on epithelial cells. J Clin Invest 2008; 118: 1657–1668. 18414680 10.1172/JCI34487 PMC2293335

[bib9] Tabas I. Consequences and therapeutic implications of macrophage apoptosis in atherosclerosis: the importance of lesion stage and phagocytic efficiency. Arterioscler Thromb Vasc Biol 2005; 25: 2255–2264. 16141399 10.1161/01.ATV.0000184783.04864.9f

[bib10] Kimani SG, Geng K, Kasikara C, Kumar S, Sriram G, Wu Y et al. Contribution of defective PS recognition and efferocytosis to chronic inflammation and autoimmunity. Front Immunol 2014; 5: 566. 25426118 10.3389/fimmu.2014.00566 PMC4226236

[bib11] Recarte-Pelz P, Tàssies D, Espinosa G, Hurtado B, Sala N, Cervera R et al. Vitamin K-dependent proteins GAS6 and protein S and TAM receptors in patients of systemic lupus erythematosus: correlation with common genetic variants and disease activity. Arthritis Res Ther 2013; 15: R41. 23497733 10.1186/ar4199 PMC3672795

[bib12] Wigren M, Nilsson J, Kaplan MJ. Pathogenic immunity in systemic lupus erythematosus and atherosclerosis: common mechanisms and possible targets for intervention. J Intern Med 2015; 278: 494–506. 25720452 10.1111/joim.12357 PMC4550575

[bib13] Thorp E, Cui D, Schrijvers DM, Kuriakose G, Tabas I. Mertk receptor mutation reduces efferocytosis efficiency and promotes apoptotic cell accumulation and plaque necrosis in atherosclerotic lesions of apoe-/- mice. Arterioscler Thromb Vasc Biol 2008; 28: 1421–1428. 18451332 10.1161/ATVBAHA.108.167197 PMC2575060

[bib14] Thorp E, Tabas I. Mechanisms and consequences of efferocytosis in advanced atherosclerosis. J Leukoc Biol 2009; 86: 1089–1095. 19414539 10.1189/jlb.0209115 PMC2774877

[bib15] Foks AC, Engelbertsen D, Kuperwaser F, Alberts-Grill N, Gonen A, Witztum JL et al. Blockade of Tim-1 and Tim-4 enhances atherosclerosis in low-density lipoprotein receptor-deficient mice. Arterioscler Thromb Vasc Biol 2016; 36: 456–465. 26821944 10.1161/ATVBAHA.115.306860 PMC4853762

[bib16] Van Vré EA, Ait-Oufella H, Tedgui A, Mallat Z. Apoptotic cell death and efferocytosis in atherosclerosis. Arterioscler Thromb Vasc Biol 2012; 32: 887–893. 22328779 10.1161/ATVBAHA.111.224873

[bib17] Dalli J, Consalvo AP, Ray V, Di Filippo C, D'Amico M, Mehta N et al. Proresolving and tissue-protective actions of annexin A1-based cleavage-resistant peptides are mediated by formyl peptide receptor 2/lipoxin A4 receptor. J Immunol 2013; 190: 6478–6487. 23686496 10.4049/jimmunol.1203000

[bib18] Flannagan RS, Jaumouillé V, Grinstein S. The cell biology of phagocytosis. Annu Rev Pathol 2011; 7: 61–98. 21910624 10.1146/annurev-pathol-011811-132445

[bib19] Kinchen JM, Doukoumetzidis K, Almendinger J, Stergiou L, Tosello-Trampont A, Sifri CD et al. A pathway for phagosome maturation during engulfment of apoptotic cells. Nat Cell Biol 2008; 10: 556–566. 18425118 10.1038/ncb1718 PMC2851549

[bib20] Kitano M, Nakaya M, Nakamura T, Nagata S, Matsuda M. Imaging of Rab5 activity identifies essential regulators for phagosome maturation. Nature 2008; 453: 241–245. 18385674 10.1038/nature06857

[bib21] Kinchen JM, Ravichandran KS. Identification of two evolutionarily conserved genes regulating processing of engulfed apoptotic cells. Nature 2010; 464: 778–782. 20305638 10.1038/nature08853 PMC2901565

[bib22] Rink J, Ghigo E, Kalaidzidis Y, Zerial M. Rab conversion as a mechanism of progression from early to late endosomes. Cell 2005; 122: 735–749. 16143105 10.1016/j.cell.2005.06.043

[bib23] Epp N, Rethmeier R, Krämer L, Ungermann C. Membrane dynamics and fusion at late endosomes and vacuoles – Rab regulation, multisubunit tethering complexes and SNAREs. Eur J Cell Biol 2011; 90: 779–785. 21683469 10.1016/j.ejcb.2011.04.007

[bib24] Harrison RE, Bucci C, Vieira OV, Schroer TA, Grinstein S. Phagosomes fuse with late endosomes and/or lysosomes by extension of membrane protrusions along microtubules: role of Rab7 and RILP. Mol Cell Biol 2003; 23: 6494–6506. 12944476 10.1128/MCB.23.18.6494-6506.2003 PMC193691

[bib25] Johansson M, Rocha N, Zwart W, Jordens I, Janssen L, Kuijl C et al. Activation of endosomal dynein motors by stepwise assembly of Rab7-RILP-p150Glued, ORP1L, and the receptor??III spectrin. J Cell Biol 2007; 176: 459–471. 17283181 10.1083/jcb.200606077 PMC2063981

[bib26] Cantalupo G, Alifano P, Roberti V, Bruni CB, Bucci C. Rab-interacting lysosomal protein (RILP): the Rab7 effector required for transport to lysosomes. EMBO J 2001; 20: 683–693. 11179213 10.1093/emboj/20.4.683 PMC145419

[bib27] Saric A, Hipolito VEB, Kay JG, Canton J, Antonescu CN, Botelho RJ. mTOR controls lysosome tubulation and antigen presentation in macrophages and dendritic cells. Mol Biol Cell 2016; 27: 321–333. 26582390 10.1091/mbc.E15-05-0272 PMC4713134

[bib28] Chakraborty D, Banerjee S, Sen A, Banerjee KK, Das P, Roy S. *Leishmania donovani* affects antigen presentation of macrophage by disrupting lipid rafts. J Immunol 2005; 175: 3214–3224. 16116212 10.4049/jimmunol.175.5.3214

[bib29] Meier CL, Svensson M, Kaye PM. Leishmania-induced inhibition of macrophage antigen presentation analyzed at the single-cell level. J Immunol 2003; 171: 6706–6713. 14662874 10.4049/jimmunol.171.12.6706

[bib30] Martinez-Pomares L, Gordon S. Antigen presentation the macrophage way. Cell 2007; 131: 641–643. 18022354 10.1016/j.cell.2007.10.046

[bib31] Von Knethen A, Sha LK, Kuchler L, Heeg AK, Fuhrmann D, Heide H et al. 5-Lipoxygenase contributes to PPARg activation in macrophages in response to apoptotic cells. Cell Signal 2013; 25: 2762–2768. 24036216 10.1016/j.cellsig.2013.08.045

[bib32] Johann AM, von Knethen A, Lindemann D, Brüne B. Recognition of apoptotic cells by macrophages activates the peroxisome proliferator-activated receptor-gamma and attenuates the oxidative burst. Cell Death Differ 2006; 13: 1533–1540. 16341123 10.1038/sj.cdd.4401832

[bib33] Yoon Y-S, Kim S-Y, Kim M-J, Lim J-H, Cho M-S, Kang JL. PPARγ activation following apoptotic cell instillation promotes resolution of lung inflammation and fibrosis via regulation of efferocytosis and proresolving cytokines. Mucosal Immunol 2015; 8: 1031–1046. 25586556 10.1038/mi.2014.130 PMC4762910

[bib34] Das A, Ganesh K, Khanna S, Sen CK, Roy S. Engulfment of apoptotic cells by macrophages: a role of MicroRNA-21 in the resolution of wound inflammation. J Immunol 2014; 192: 1120–1129. 24391209 10.4049/jimmunol.1300613 PMC4358325

[bib35] Mondal S, Ghosh-Roy S, Loison F, Li Y, Jia Y, Harris C et al. PTEN negatively regulates engulfment of apoptotic cells by modulating activation of Rac GTPase. J Immunol 2011; 187: 5783–5794. 22043008 10.4049/jimmunol.1100484 PMC3221816

[bib36] Zizzo G, Hilliard BA, Monestier M, Cohen PL. Efficient clearance of early apoptotic cells by human macrophages requires M2c polarization and MerTK induction. J Immunol 2012; 189: 3508–3520. 22942426 10.4049/jimmunol.1200662 PMC3465703

[bib37] Lavine KJ, Epelman S, Uchida K, Weber KJ, Nichols CG, Schilling JD et al. Distinct macrophage lineages contribute to disparate patterns of cardiac recovery and remodeling in the neonatal and adult heart. Proc Natl Acad Sci USA 2014; 111: 16029–16034. 25349429 10.1073/pnas.1406508111 PMC4234568

[bib38] Boraschi D. Transcriptomic profiling of the development of the inflammatory response in human monocytes *in vitro*. PLoS One 2014; 9: e87680. 24498352 10.1371/journal.pone.0087680 PMC3912012

[bib39] Martinez FO, Gordon S, Locati M, Mantovani A. Transcriptional profiling of the human monocyte-to-macrophage differentiation and polarization: new molecules and patterns of gene expression. J Immunol 2006; 177: 7303–7311. 17082649 10.4049/jimmunol.177.10.7303

[bib40] Arnold CE, Gordon P, Barker RN, Wilson HM. The activation status of human macrophages presenting antigen determines the efficiency of Th17 responses. Immunobiology 2015; 220: 10–19. 25454489 10.1016/j.imbio.2014.09.022

[bib41] Beaumont KA, Hamilton NA, Moores MT, Brown DL, Ohbayashi N, Cairncross O et al. The recycling endosome protein Rab17 regulates melanocytic filopodia formation and melanosome trafficking. Traffic 2011; 12: 627–643. 21291502 10.1111/j.1600-0854.2011.01172.x

[bib42] Zacchi P, Stenmark H, Parton RG, Orioli D, Lim F, Giner A et al. Rab17 regulates membrane trafficking through apical recycling endosomes in polarized epithelial cells. J Cell Biol 1998; 140: 1039–1053. 9490718 10.1083/jcb.140.5.1039 PMC2132691

[bib43] Hunziker W, Peters PJ. Rab17 localizes to recycling endosomes and regulates receptor-mediated transcytosis in epithelial cells. J Biol Chem 1998; 273: 15734–15741. 9624171 10.1074/jbc.273.25.15734

[bib44] Shintani M, Tada M, Kobayashi T, Kajiho H, Kontani K, Katada T. Characterization of Rab45/RASEF containing EF-hand domain and a coiled-coil motif as a self-associating GTPase. Biochem Biophys Res Commun 2007; 357: 661–667. 17448446 10.1016/j.bbrc.2007.03.206

[bib45] Reboulet RA, Hennies CM, Garcia Z, Nierkens S, Janssen EM. Prolonged antigen storage endows merocytic dendritic cells with enhanced capacity to prime anti-tumor responses in tumor-bearing mice. J Immunol 2010; 185: 3337–3347. 20720209 10.4049/jimmunol.1001619 PMC3021914

[bib46] Striz AC, Tuma PL. The GTP-bound and sumoylated form of the RAB17 small molecular weight GTPase selectively binds syntaxin 2 in polarized hepatic WIF-B cells. J Biol Chem 2016; 291: 9721–9732. 26957544 10.1074/jbc.M116.723353 PMC4850309

[bib47] Mori Y, Matsui T, Fukuda M. Rabex-5 protein regulates dendritic localization of small GTPase Rab17 and neurite morphogenesis in hippocampal neurons. J Biol Chem 2013; 288: 9835–9847. 23430262 10.1074/jbc.M112.427591 PMC3617284

[bib48] Mori Y, Fukuda M. Assay of Rab17 and its guanine nucleotide exchange factor Rabex-5 in the dendrites of hippocampal neurons. Methods Mol Biol 2015; 1298: 233–243. 25800847 10.1007/978-1-4939-2569-8_20

[bib49] Haobam B, Nozawa T, Minowa-Nozawa A, Tanaka M, Oda S, Watanabe T et al. Rab17-mediated recycling endosomes contribute to autophagosome formation in response to Group A *Streptococcus* invasion. Cell Microbiol 2014; 16: 1806–1821. 25052408 10.1111/cmi.12329

[bib50] Minakami R, Maehara Y, Kamakura S, Kumano O, Miyano K, Sumimoto H. Membrane phospholipid metabolism during phagocytosis in human neutrophils. Genes Cells 2010; 15: 409–424. 20384786 10.1111/j.1365-2443.2010.01393.x

[bib51] Horiuchi H, Lippé R, McBride HM, Rubino M, Woodman P, Stenmark H et al. A novel Rab5 GDP/GTP exchange factor complexed to Rabaptin-5 links nucleotide exchange to effector recruitment and function. Cell 1997; 90: 1149–1159. 9323142 10.1016/s0092-8674(00)80380-3

[bib52] Chen D, Xiao H, Zhang K, Wang B, Gao Z, Jian Y et al. Retromer is required for apoptotic cell clearance by phagocytic receptor recycling. Science 2010; 327: 1261–1264. 20133524 10.1126/science.1184840

[bib53] Malbran A, Siwik S, Frank MM, Fries LF. CR1-receptor recycling in phorbol ester-activated polymorphonuclear leucocytes. Immunology 1988; 63: 325–330. 3162434 PMC1454518

[bib54] Cox D, Lee DJ, Dale BM, Calafat J, Greenberg S. A Rab11-containing rapidly recycling compartment in macrophages that promotes phagocytosis. Proc Natl Acad Sci USA 2000; 97: 680–685. 10639139 10.1073/pnas.97.2.680 PMC15390

[bib55] Moore RH, Millman EE, Alpizar-Foster E, Dai W, Knoll BJ. Rab11 regulates the recycling and lysosome targeting of beta2-adrenergic receptors. J Cell Sci 2004; 117: 3107–3117. 15190120 10.1242/jcs.01168

[bib56] Savill J, Hogg N, Ren Y, Haslett C. Thrombospondin cooperates with CD36 and the vitronectin receptor in macrophage recognition of neutrophils undergoing apoptosis. J Clin Invest 1992; 90: 1513–1522. 1383273 10.1172/JCI116019 PMC443198

[bib57] Caberoy NB, Alvarado G, Bigcas J-L, Li W. Galectin-3 is a new MerTK-specific eat-me signal. J Cell Physiol 2012; 227: 401–407. 21792939 10.1002/jcp.22955 PMC3225605

[bib58] Poteryaev D, Datta S, Ackema K, Zerial M, Spang A. Identification of the switch in early-to-late endosome transition. Cell 2010; 141: 497–508. 20434987 10.1016/j.cell.2010.03.011

[bib59] Ejlerskov P, Christensen DP, Beyaie D, Burritt JB, Paclet MH, Gorlach A et al. NADPH oxidase is internalized by clathrin-coated pits and localizes to a Rab27A/B GTPase-regulated secretory compartment in activated macrophages. J Biol Chem 2012; 287: 4835–4852. 22157766 10.1074/jbc.M111.293696 PMC3281610

[bib60] Wang H, Ishizaki R, Xu J, Kasai K, Kobayashi E, Gomi H et al. The Rab27a effector exophilin7 promotes fusion of secretory granules that have not been docked to the plasma membrane. Mol Biol Cell 2013; 24: 319–330. 23223571 10.1091/mbc.E12-04-0265 PMC3564536

[bib61] Elstak ED, Neeft M, Nehme NT, Callebaut I, de Saint Basile G, van der Sluijs P. Munc13-4*rab27 complex tethers secretory lysosomes at the plasma membrane. Commun Integr Biol 2012; 5: 64–67. 22482013 10.4161/cib.18015 PMC3291317

[bib62] Vaux DL, Korsmeyer SJ. Cell death in development. Cell 1999; 96: 245–254. 9988219 10.1016/s0092-8674(00)80564-4

[bib63] Binder MD, Fox AD, Merlo D, Johnson LJ, Giuffrida L, Calvert SE et al. Common and low frequency variants in MERTK are independently associated with multiple sclerosis susceptibility with discordant association dependent upon HLA-DRB1*15:01 status. PLoS Genet 2016; 12: e1005853. 26990204 10.1371/journal.pgen.1005853 PMC4798184

[bib64] Kenyon KD, Cole C, Crawford F, Kappler JW, Thurman JM, Bratton DL et al. IgG autoantibodies against deposited C3 inhibit macrophage-mediated apoptotic cell engulfment in systemic autoimmunity. J Immunol 2011; 187: 2101–2111. 21813769 10.4049/jimmunol.1003468 PMC3159788

[bib65] Heo K-S, Cushman HJ, Akaike M, Woo C-H, Wang X, Qiu X et al. ERK5 activation in macrophages promotes efferocytosis and inhibits atherosclerosis. Circulation 2014; 130: 180–191. 25001623 10.1161/CIRCULATIONAHA.113.005991 PMC4439099

[bib66] Spinner JL, Winfree S, Starr T, Shannon JG, Nair V, Steele-Mortimer O et al. Yersinia pestis survival and replication within human neutrophil phagosomes and uptake of infected neutrophils by macrophages. J Leukoc Biol 2013; 95: 1–10. 10.1189/jlb.1112551 PMC3923079 24227798

[bib67] Walseng E, Furuta K, Bosch B, Weih KA, Matsuki Y, Bakke O et al. Ubiquitination regulates MHC class II-peptide complex retention and degradation in dendritic cells. Proc Natl Acad Sci USA 2010; 107: 20465–20470. 21059907 10.1073/pnas.1010990107 PMC2996684

[bib68] Cho K-J, Walseng E, Ishido S, Roche PA. Ubiquitination by March-I prevents MHC class II recycling and promotes MHC class II turnover in antigen-presenting cells. Proc Natl Acad Sci USA 2015; 112: 10449–10454. 26240324 10.1073/pnas.1507981112 PMC4547296

[bib69] Evans A, Blackburn J, Yin C, Heit B. Quantitative efferocytosis assays. Methods Mol Biol Phagocytosis Phagosome Matur 2017; 1519: 24–41. 10.1007/978-1-4939-6581-6_3 27815871

[bib70] Smith AL, Friedman DB, Yu H, Carnahan RH, Reynolds AB. ReCLIP (reversible cross-link immuno-precipitation): an efficient method for interrogation of labile protein complexes. PLoS One 2011; 6: e16206. 21283770 10.1371/journal.pone.0016206 PMC3024417

[bib71] Azizi PM, Zyla RE, Guan S, Wang C, Liu J, Bolz S-S et al. Clathrin-dependent entry and vesicle-mediated exocytosis define insulin transcytosis across microvascular endothelial cells. Mol Biol Cell 2014; 26: 740–750. 25540431 10.1091/mbc.E14-08-1307 PMC4325843

[bib72] Armstrong SM, Sugiyama MG, Fung KYY, Gao Y, Wang C, Levy AS et al. A novel assay uncovers an unexpected role for SR-BI in LDL transcytosis. Cardiovasc Res 2015; 108: 268–277. 26334034 10.1093/cvr/cvv218 PMC4614686

[bib73] Pillon NJ, Azizi PM, Li YE, Liu J, Wang C, Chan KL et al. Palmitate-induced inflammatory pathways in human adipose microvascular endothelial cells promote monocyte adhesion and impair insulin transcytosis. Am J Physiol Endocrinol Metab 2015; 309: E35–E44. 25944880 10.1152/ajpendo.00611.2014

[bib74] Crocker JC, Grier DG. Methods of digital video microscopy for colloidal studies. J Colloid Interface Sci 1996; 179: 298–310.

