# Supplementary material for: Rab17 mediates differential antigen sorting following efferocytosis and phagocytosis
Source: Cell Death Dis. 2016 Dec 22;7(12):e2529–. doi: 10.1038/cddis.2016.431 (PMC5261003; doi:10.1038/cddis.2016.431)
Supplement: Supplementary Figures [file cddis2016431x1.pdf]

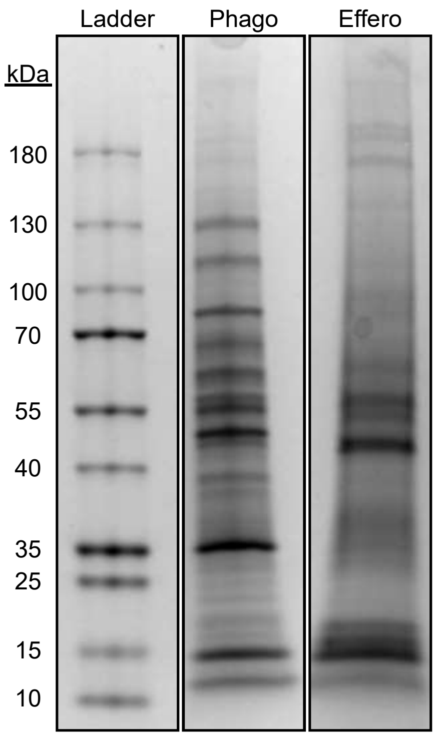

Supplemental Figure 1: Efferosomes and Phagosomes Interact with Unique Subsets of Proteins. Coomassie staining of SDS-PAGE gels of proteins recovered from phagosomes (phago) and efferosomes (effero) 40 min following efferocytosis or phagocytosis. Efferosomes and phagosomes were recovered from human PBMC-derived M0 macrophages.

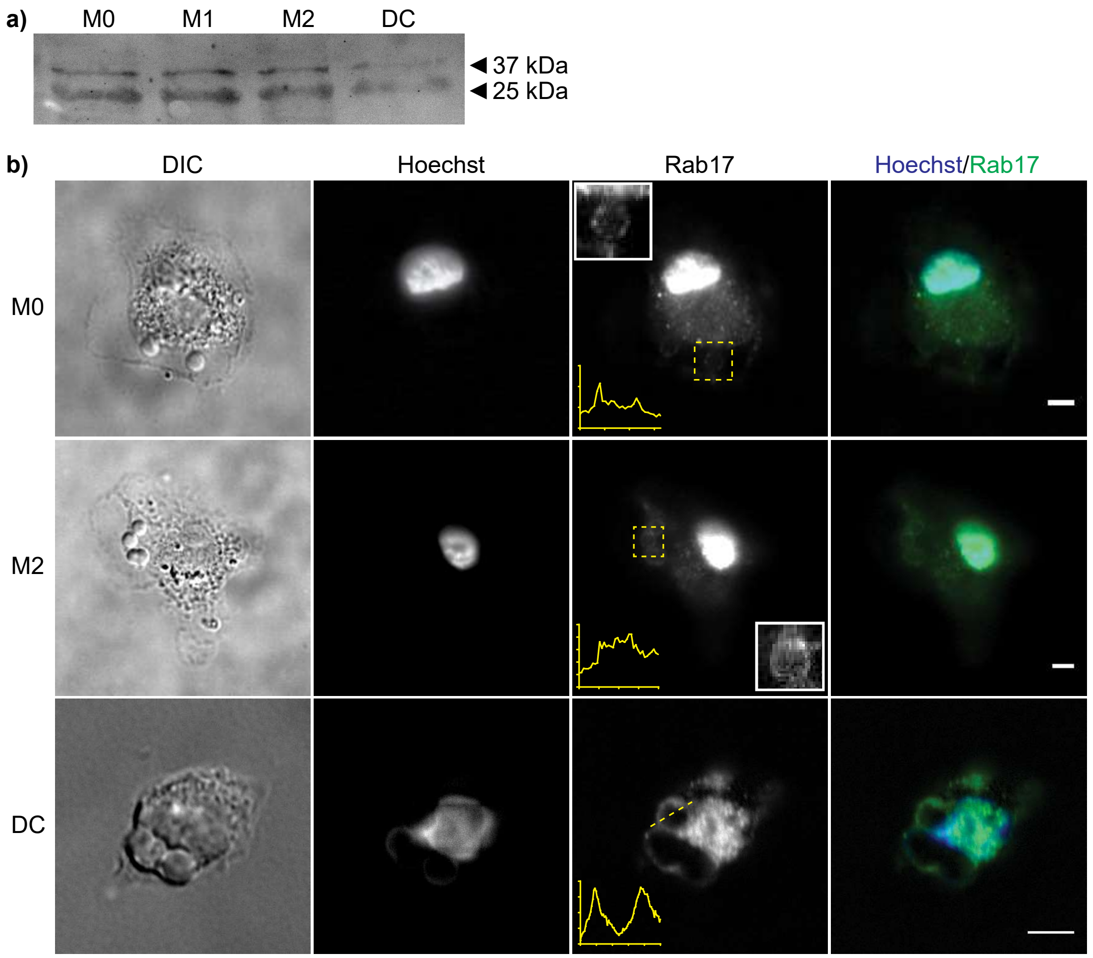

Supplemental Figure 2: Rab17 is Expressed in Human Phagocytes and is Recruited to Efferosomes Late in Maturation. Macrophages and dendritic cells were derived by *ex vivo* differentiation of human peripheral blood mononuclear cells into the respective cell types. **a)** Rab17 immunoblot of M0, M1 and M2-polarized macrophages and dendritic cells (DC). Both the sumoylated (~37 kDa) and unsumoylated (~25 kDa) forms of Rab17 are present in all four cell types. **b)** Immunostaining of Rab17 30 min following efferosome closure. Plots indicate the intensity of Rab17 staining along the dashed yellow line (DC) or horizontally across the center of the insert (M0/M2). M1-polarized macrophages are not presented as no efferosomes were observed in these cells. Images are representative of 3 independent experiments, scale bars are 5 μm.

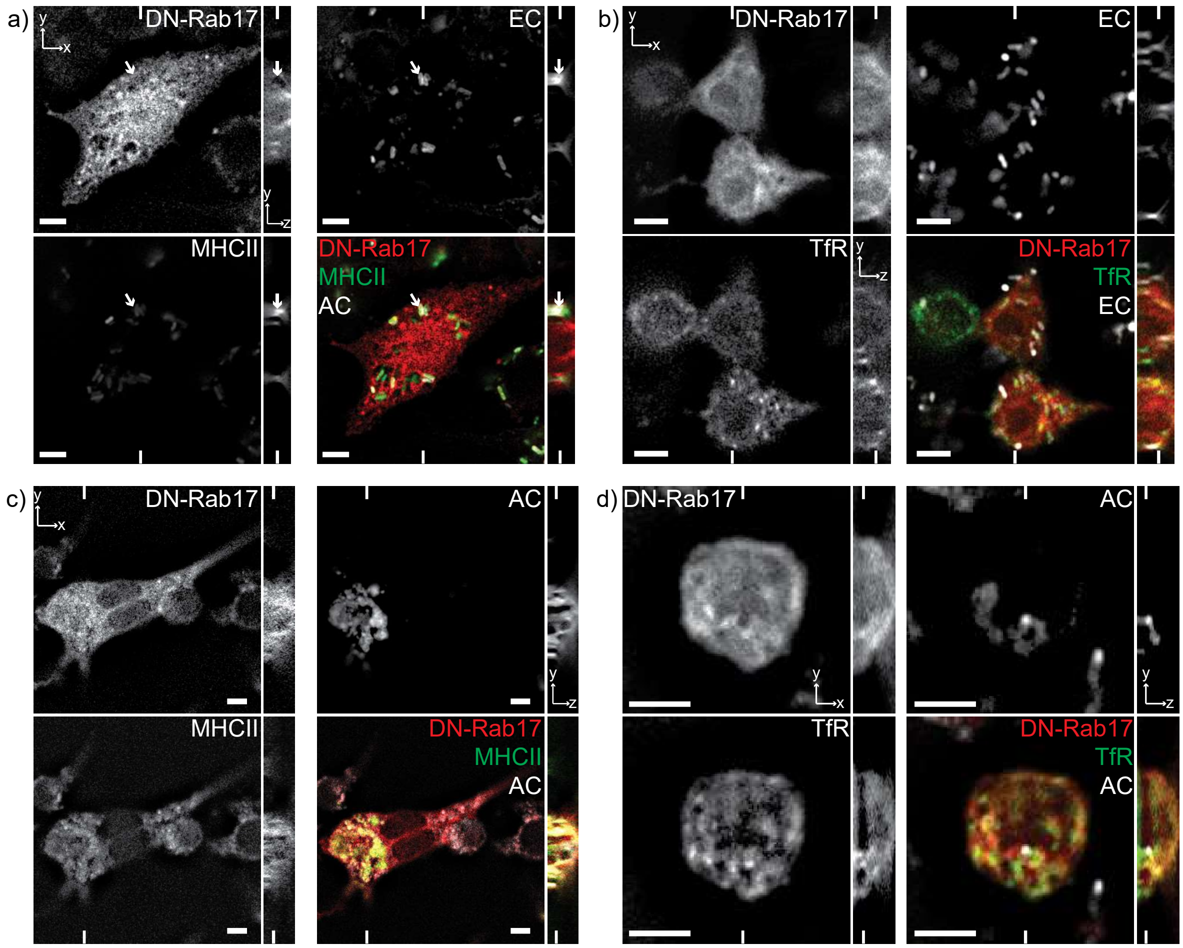

Supplemental Figure 3: Effects of Dominant-Negative Rab17 on MHC II and TfR Recruitment to Efferosomes and Phagosomes. Fixed cell microscopy was performed on J774.2 macrophages expressing dominant-negative Rab17-mCherry (DN-Rab17) and either co-expressing transferrin receptor-GFP (TfR-GFP) or immunostained for MHC II. a-d) Localization of DN-Rab17 and MHC II (a,c) or TfR (b,d), relative to phagocytosed E. coli (a,b) or efferocytosed apoptotic cells (c,d). Images are representative of a minimum of 15 images captured in 3 independent experiments. Scale bars are 5 µm, small lines indicate the position of the cross-section shown in the corresponding xy and z images.
